# Societal Perceptions and Understanding of Voyeurism & Upskirting in Young Adult Singaporean Nationals: A Reflexive Thematic Analysis

**DOI:** 10.3390/bs16040531

**Published:** 2026-04-01

**Authors:** Alfeera Natasha Jumat, Georgina Mclocklin, Dean Fido

**Affiliations:** School of Science, University of Derby, Kedleston Road, Derbyshire, Derby DE22 1GB, UK; alfeeranatasha@gmail.com (A.N.J.); g.mclocklin@derby.ac.uk (G.M.)

**Keywords:** voyeurism, upskirting, technology-facilitated sexual violence, gendered crime, image-based sexual abuse

## Abstract

Despite near-global legal reforms to tackle voyeurism and upskirting offences (VUs), such behaviours remain prevalent in Singapore—an under-reached population for empirical research in the niche of image-based sexual abuse and one where conservative views and sex-related taboos persist. This study consists of interviews with ten young adult Singaporean nationals about their understanding of VUs, victim-survivors thereof, and how such views interact with Singaporean culture and societal norms. Reflective thematic analysis was used to delineate the two predominant themes of the (1) *Unaccountability of Perpetrators*, wherein VUs are minimised and excused at both societal (*Technological and Institutional Affordances*) and individual levels (*Sexual Deviancy & Pornography*), and (2) *Burden of Victimisation*, which explored perceptions of victim-survivors as a gendered experience (*Gendered Vulnerability*), where norms around modesty impacted victim-blaming (*Moralised Modesty & Responsibility*), resulting in harm minimisation (*Harm Awareness & Minimisation*). Findings have implications for how legislators, law enforcement, and educational institutions address the minimisation of gender-based violence through shifts in social narratives, awareness, and responses.

## 1. Introduction

Image-based sexual abuse (IBSA) encompasses the unsolicited sending (e.g., cyber-flashing), creation (e.g., deepfake sexual abuse), non-consensual sharing (e.g., ‘revenge pornography’^1^) and capturing (e.g., upskirting) of intimate media (Fido & Harper, 2020; McGlynn et al., 2017). Under the United Kingdom’s Sexual Offences Act 2003 and Voyeurism (Offences) Act 2019, voyeurism refers to acts whereby one gains sexual gratification from observing or recording someone engaged in private activity without consent, with upskirting specifically referring to using equipment to take photos or videos underneath one’s clothing without consent (Fido et al., 2025; Lister & Gannon, 2024; Wdowiak et al., 2025). Voyeuristic and upskirting offences (VUs) are legislated against across most of the developed world and are considered societal issues requiring integrated attention from clinicians, researchers, and criminal justice systems (Hocken & Thorne, 2012; Lister & Gannon, 2024); however, said legislation varies among countries and states therein.

Singapore represents an under-reached context for empirically understanding VUs, which are governed compositely under the Criminal Law Reform Act 2019 (s 377BB). This legislation employs a consent-focused lens that was absent in previous governance of VUs, which instead centred around whether a victim’s modesty was deemed to have been “insulted” (Singapore Statutes Online, 2008, s 509) and which focused on public morals and societal protection from explicit material (Singapore Statutes Online, 1998). However, the Criminal Law Reform Act 2019 still fails to address the multi-faceted nature and manifestations of VUs, as well as the underpinning motivations to capture, distribute, and/or threaten to distribute intimate media (Vitis, 2021a). This is important, as despite Singapore ranking as the fifth safest country globally (Global Peace Index, 2024) and having a reputation for high safety and low physical crime rates (331 cases per 100,000 individuals; Singapore Department of Statistics, 2025), VUs increased by 9% between 2023 and 2024 in Singapore and only narrowly decreased in 2025 (Singapore Police Force, 2024, 2025). Amplifying this harm are reports of the widespread circulation of explicit sexual images, including hidden camera footage and upskirting images within encrypted Singapore-based social media groups (e.g., SharingisCaring, SGGirls@SGWikiLeaks; Mann, 2022).

A pertinent Singaporean-based case featuring VUs is that of Monica Baey, who was non-consensually filmed showering by a fellow undergraduate student at the National University of Singapore (Ting, 2019b). S. Tan’s (2023) examination of social media discourse on this case detailed victim-blaming comments (e.g., “…they need to also understand n accept how a man think”, p. 65) and those which minimised the offence (e.g., “… at least he’s not out raping women”, p. 67). Views which map onto du Mello Gibbard and Fido’s (2023) findings that physical sexual offences are perceived to be more serious than IBSA, and Harper et al.’s (2023) conceptualisation of societal beliefs about the non-consensual sharing of intimate images (NCSII).

The importance of understanding how such judgements manifest is many-fold. From a victim-survivor perspective, experiencing and anticipating victim-blaming or harm-minimising reactions can prevent reporting and seeking support (Adair, 2022; Mclocklin et al., 2025), especially in regions with low levels of support services for women and girls (Rousaki & Fido, 2026). Though women in Singapore have access to relevant services (e.g., AWARE Singapore; Singapore Council of Women’s Organisations), engaging with support remains vital given that victim-survivors of IBSA face several professional- (e.g., loss of employment), social- (e.g., isolation, shame), and health-related (e.g., depression, suicidal ideations) consequences (Bates, 2017; Fido & Harper, 2020; Hall et al., 2023). From a legal standpoint, understanding how society views VUs impacts the identification of perpetrators and their subsequent evaluation by those within the criminal justice system, such as police and juries (Fido et al., 2024, 2025, 2026). Though this literature largely derives from Westernised cultures, in a Singaporean context, victim-blaming can be partially explained through the high valuation placed on modesty and social acceptability (Koh & Wang, 2012), which, for women, is achieved by dressing conservatively, wearing minimal accessories, and avoiding unwanted male attention (Chen et al., 2009; Yeoh & Yeow, 1997). Within this context, being a victim of VUs might imply that one ‘failed’ to conform to gendered-norms and that they disengaged from self-protection strategies; effectively shifting blame onto them and away from the perpetrators. This victim-survivor focus was reflected in Lam and Lau’s (2024) report on VUs in Singapore, wherein victim-survivors collectively disclosed changing their behaviour as a result of victimisation, including wearing extra layers of clothing and standing sideways on escalators. These participants also expressed an array of psychological impacts in the forms of anxiety, paranoia, and suspicion when in public places.

On a theoretical level, the prevailing model of VUs is Lister and Gannon’s (2024) Descriptive Model of Voyeuristic Behavior, which outlines how ones’ propensity to engage in voyeuristic behaviour can be predicted through variation in affective, behavioural, cognitive, and contextual factors, wherein negative early experiences (e.g., peer and/or intimate relationships) contribute to antisocial and atypical sexual behaviour, which, in turn, interact with experiences of life dissatisfaction and poor social support. When met with risk factors such as hypersexuality, sexual habituation, poor mental health, and external pressures, pathways to engagement in VUs may be explained as a function of gaining *Sexual Gratification*, having *Maladaptive Coping Strategies*, and wanting *Access to Inappropriate Person(s)*. Such pathways might be contributed to through pornography consumption, by reinforcing maladaptive sexual scripts (Hopkins et al., 2016; Seto et al., 2001), a facet associated with engagement in voyeurism, broadly (Langström & Seto, 2006) and within a Singaporean context, specifically (Ting, 2019a).

### Current Study

With research on IBSA being predominantly situated within Western contexts, and with VUs remaining comparatively under-researched relative to other forms (e.g., NCSII, deepfake sexual abuse), this study seeks to explore Singaporean narratives regarding the perceptions of VUs in a country where such offences are both prevalent and governed with limited nuance. In doing so, it explores perceptions of VUs as criminal offences, implications on victim-survivor groups, and, ultimately, seeks to understand how our sample constructs motivations to engage in VUs. As such, the study aimed to investigate the following research question: How do young adults in Singapore perceive VUs, their impact, and victim-survivors thereof, and what, if anything, do they consider motivations to engage in such behaviour?

## 2. Methods

### 2.1. Design

This study adopted a qualitative design using semi-structured interviews, which enabled an inductive and exploratory examination of how cultural norms shape attitudes towards VUs and victim-survivors thereof. This approach is suited to capturing the nuance, complexity and contextual specificity of participants’ experiences and meanings (Braun & Clarke, 2022), which are essential for understanding how VUs are perceived within the Singaporean sociocultural landscape.

### 2.2. Participants

Ten participants (*n* = 6 females; *n* = 4 males; age range = 26–33 years; M_age_ = 29.2 years) were interviewed after being recruited through a social media campaign. Participants had to be aged 18 years or older, fluent in English to meet local University ethical requirements, and both of Singaporean heritage and residency to control for variation in culture and legal knowledge.

In line with Reflexive Thematic Analysis (RTA), sample adequacy is not determined by numerical thresholds or by achieving data saturation, which is conceptually incompatible with RTA’s epistemological stance (Braun & Clarke, 2021). Instead, adequacy is evaluated through the richness and depth of the data and the degree of information power present within the sample (Malterud et al., 2016). Given the study’s aims, the moderately focused population (young Singaporean adults who use social media and who are fluent in English), and the richness of the interviews, the sample (see Table 1) provided sufficient information power to develop coherent, meaningful themes within this analytic framework.

## 3. Procedure and Materials

The study was approved by an ethics committee of the University of Derby [Ref: ETH2425-1795]. Participants were directed to an information sheet, consent form, and interview booking form hosted via Qualtrics and were asked to report their age, gender, and nationality, and self-select a pseudonym. Between 6 February and 1 March 2025, semi-structured interviews were conducted (in English), recorded, and transcribed verbatim via Microsoft Teams, which lasted between 45 and 60 min.

The interview schedule centred around (i) general knowledge pertaining to VUs, (ii) perceptions of individuals who perpetrate VUs and their motivations, (iii) perceptions of the victim-survivors of VUs and associated impacts, and [iv] the role of social and cultural contexts in Singapore in said answers. The interview scheduled used open-ended questions to facilitate more in-depth discussions. Afterwards, participants were given the opportunity to add any further information they wished that was not elicited directly via the interview, before being verbally debriefed.

### 3.1. Data Analysis

Interviews were analysed using RTA to develop themes that captured patterns across the data (Braun & Clarke, 2022). A critical realist lens was adopted, which assumes there is an objective reality; however, our interpretation of such, is subjective and influenced by social–cultural constructs (Lawani, 2021). The analysis followed Braun and Clarke’s (2022) six-step process, which involved the following: (1) engaging with the data and making initial annotations on each transcript, (2) generating initial codes relevant to the research questions, (3) constructing themes via grouping the initial codes to capture patterns in the data, (4) collectively reviewing themes to ensure accuracy, (5) refining and labelling the themes to reflect their core meanings, and (6) generating a coherent structure and narrative for the themes. The interpretative nature of this approach facilitated comprehension, description, and explanation of VUs from the perspectives of the individual participants and their position within society (Tong et al., 2007); enabling a deeper understanding of perceptions and beliefs within a Singaporean context.

### 3.2. Reflexivity

The research team approached the research from a forensic psychological background. The first author, who conducted and analysed all the interviews, identifies as a Singaporean woman, born and raised in Singapore. Her cultural familiarity and personal understanding of social norms gave her an insider perspective that shaped both data generation and interpretation. For example, sharing a cultural background with participants may have helped establish rapport and enabled them to feel more comfortable discussing their culture more openly by assuming a shared understanding.

The project was supervised by the second and third authors, both British nationals with specialist knowledge on IBSA and greater experience in qualitative research methods. These authors contributed to the analysis by engaging in discussions that facilitated theme development and informed the writing process through their topic and theoretical expertise.

## 4. Findings and Discussion

**Figure 1:**
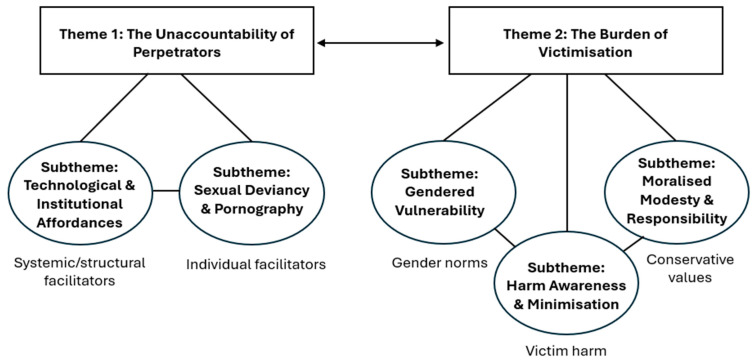
Thematic map of themes and subthemes.

The following two main themes were developed: (1) *the unaccountability of offenders* explored and (2) *the burden of victimisation.* Figure 1 presents a thematic map to show the relationship between the themes and subthemes.

### 4.1. Theme 1: The Unaccountability of Perpetrators

The first theme explored how VUs are minimised and excused at both societal (subtheme 1.1) and individual levels (subtheme 1.2), resulting in perpetrators being perceived as unaccountable.

#### 4.1.1. Subtheme 1.1 Technological and Institutional Affordances

Participants highlighted how broadly there was little knowledge surrounding the legislation of consequences of VUs, with institutional responses doing little to educate Singaporean society:

“*…Government don’t really share much on how serious the consequences of upskirting are…if you take drugs or sell drugs, it’s widely shared across… but for upskirting… it’s not shared what will happen to you…*”

(Mahmat, 30)

Mahmat discussed how, at the institutional level, VUs are not taken as seriously as other offences, supporting research suggesting that physical offences are treated more seriously (du Mello Gibbard & Fido, 2023). Mahmat noted that this institutional minimisation inhibits the public sharing of legislative knowledge, resulting in an uninformed society, whereby this minimisation is reflected more widely in the public. Additionally, as certainty of punishment deters crime more effectively than the severity of punishment (Nagin, 2013), this lack of knowledge and certainty of prosecution does little to prevent VUs and thus may contribute to a cultural tolerance of VUs. Participants noted the lack of repercussions for perpetrators could be further exacerbated by the technology-facilitated nature of VUs:

“*… You have apps like Telegram where you can form a group without even putting your own name and circulate all these pictures around without getting caught.*”

(Jean, 32)

Jean reflected on how anonymity afforded through encrypted platforms, such as Telegram, can enable perpetrators to remain undetected, resulting in an inability to hold those who perpetrate VUs accountable, leaving victim-survivors without clear recourse (AWARE, 2023). Thus, the online nature is perceived to further reduce the certainty of punishment as perpetrators’ risk of apprehension is minimised, thereby creating the perception that offending is safe (Nagin, 2013). Collectively, these comments highlight that insufficient public messaging about prosecutions and technological features can lead to a perceived impunity of perpetrators of VUs.

In addition to removing accountability, participants noted that social media and entertainment platforms may normalise non-consensual sexual behaviours:

“*…I think it’s very like worrying when I see some of the content on… TikTok, Instagram, Twitter, where it’s like they’re romanticising not giving consent… so… from the perpetrators point of view… they think it’s okay… So I think the media is kind of making it slightly, I mean, not slightly, I think it’s just blurring the lines of consent.*”

(A.H., 26)

A.H.’s reflection shows how technology platforms may contribute to and promote the normalisation of harmful sexual scripts that may shape non-consensual behaviours as not only acceptable, but playful or desirable (‘romanticising’). This may, in part, be facilitated by the low accountability online, which can promote abusive behaviours (Kunhao et al., 2024; Lapidot-Lefler & Barak, 2012). However, Jean noted how these non-consensual behaviours, are also shown in entertainment media:

“*I think Netflix… like the show Elite*…*there are a few like, essence of… not giving consent… Like taking photos, videos of other girls, underwear and everything.*”

(Jean, 32)

Jean demonstrated how popular TV shows aimed at teenagers and young adults, portray VUs.

This promotion of non-consensual behaviour can produce ambiguity around what constitutes consensual sexual interactions, which, when normalised, can facilitate VUs (Groszhans, 2018). The portrayal of such behaviours, without showing the repercussions, could serve to minimise and normalise VUs. As sex education in Singapore promotes abstinence (Liew, 2014), individuals may be particularly reliant on online media to inform their understanding of consent and may receive little information counteracting portrayals which may normalise VUs (Ho et al., 2025).

Furthermore, the normalisation and minimisation of VUs within bystander intervention potentially blur the lines between socially acceptable and unacceptable behaviours (Mainwaring et al., 2023) and hinders community-level responsibility, thus perpetuating a culture of silence. This lack of public concern reflects broader social narratives that downplay the perceived severity of VUs and how they are viewed as crimes; risking toleration. Thus, participants felt both institutional and technological messaging contributed to reduced perpetrator accountability by minimising and normalising VUs, highlighting the need for greater societal education to ensure a greater understanding of VUs.

#### 4.1.2. Subtheme 1.2 Sexual Deviancy and Pornography

Within the context of lax institutional and technological environments that normalised VUs, participants reflected on the experiences and characteristics that further motivated certain individuals to engage in VUs. Participants frequently reported on their perception that high pornography consumption contributes to perpetration by desensitising them to voyeuristically styled media:

“*I feel like it’s the pursuit of adrenaline… for some people, if you consume copious amounts of pornography… it’s not gonna have the same effect… so they look for a new high sexually. (…) for example like in the Japanese culture whereby it’s commonplace to find videos of people doing it in like a public setting and I feel like the people who like consume this kind of pornography, eventually it leaks into their mind and it sort of coerces them into doing this kind of actions*”

(Luts, 27)

Luts positions pornography as a corruptive force, suggesting that VUs occur as an outcome of desensitisation caused by excessive use. By using addiction-style narratives, such as the pursuit of ‘adrenaline’ and seeking ‘a new high’, they frame VUs as compulsive, thrill-seeking behaviours that escalates from deviant sexual fantasies into ‘actions’ to achieve sexual gratification. The need to seek out more extreme or novel stimuli to achieve the same levels of gratification (Hanseder & Dantas, 2023), despite social, physical, or legal risks (Bagdasarov et al., 2010), suggests that perpetrators of VUs are perceived as having little control over their behaviour. Haze similarly reflected on the role of pornography consumption and thrill-seeking as motivators:

“*… The more that you see, the more I think it kinda gives them the power, the excitement, the thrill…*”

(Haze, 33)

Thus, these comments reflect the conceptualisation of deviant sexual interests and risk-taking behaviours as potential motivation for engaging in upskirting specifically (Harper et al., 2021) and are commonly reported in the wider sexual abuse literature (Langström & Seto, 2006; Levenson & Grady, 2016). The role pornography consumption has in reinforcing compulsive thoughts and engagement in VUs to achieve sexual gratification maps onto the pathway reinforcement discussed within the Descriptive Model of Voyeuristic Behavior (Lister & Gannon, 2024) and reflects broader literature around links between habitual pornography consumption and sexual habituation in the context of physical sexual offences (Langström & Seto, 2006). However, whilst Luts and Haze attributed VUs as acts rooted in sexual gratification and adrenaline seeking, others believed that pornography reinforced discriminatory attitudes towards women, the predominant targets in VUs (de Heer et al., 2021):

“*I think addiction is a really big thing because the porn that they’re watching is already degrading to women, so… Every young guy now has access to porn. And I feel like, when you watch it at a very young age, it distorts the idea of what actually is a sexual relationship… And then the videos out there are not the best for ladies?*”

(Naufal, 26)

Although Naufal similarly positions excessive pornography consumption as a contributing factor in VU offending, Naufal’s observations suggested that this instead arises from the internalisation of distorted views towards women. This aligns with feminist perspectives that position pornography as a medium to humiliate, degrade, and dehumanise women (Neufeld, 2020) and where gender-based norms portraying men as dominant and women as submissive are conveyed and reinforced (Rothman et al., 2021); thus, potentially influencing one’s own sexual behaviours (Yu et al., 2021).

### 4.2. Theme 2: The Burden of Victimisation

This second theme explored participants’ perceptions of victim-survivors, recognising how victimisation was a gendered experience (subtheme 2.1), where norms around modesty inflicted responsibility onto victim-survivors (subtheme 2.2) resulting in the significant harm they experience being minimised (subtheme 2.3).

#### 4.2.1. Subtheme 2.1 Gendered Vulnerability

Collectively, VUs were perceived to strictly target women, with narratives curated around gender norms and expectations:

“*I have yet to see any cases of men being victims of such, of voyeurism. And I would say in general, women are more susceptible…*”

(Sally, 26)

Although Sally recognised the gendered pattern of VUs, which disproportionately target women (Lewis & Anitha, 2023), her commentary of understanding this pattern reflected gendered discourse around vulnerability. Sally positions women as expected victims of voyeurism, compared to men who are invulnerable to such experiences. By reflecting on what she had ‘yet to see’, Sally’s knowledge and understanding of VUs may be shaped and informed by high-profile media cases which highlight male perpetrated voyeurism (e.g., Malay Mail, 2025) and upskirting (e.g., Lam & Lau, 2024; Singh, 2023) within Singapore.

Others similarly emphasised the gendered nature of VUs:

“*…I don’t feel a need to be afraid of what I’m wearing when I’m public. I feel like sometimes girls are wearing skirts in public, they might be conscious… whenever they’re on escalators or whenever they’re… bending down trying to grab something… I don’t feel self-conscious in my clothes when it comes to that…I don’t feel personally that I will be a victim of voyeurism…*”

(Naufal, 26)

As a man, Naufal elaborated on how his gender identity had a protective function that reduced his risk of being victimised. By highlighting the distinct freedoms afforded to women and men in public spaces, he further demonstrated the gendered nature of vulnerability. His discussion of women needing to be ‘conscious’ in these spaces suggests the need for women to remain constantly guarded and proactively aware of their vulnerability to reduce their risk of victimisation. This supports Adair’s (2022) framing of women as instruments for male pleasure suggests that males’ entitlement to visual access and objectification is normalised, while females are held responsible for preventing harm; thus, displacing accountability. The obligation, therefore, for women to be hyperaware of their surroundings presents stark implications shaped by societal and cultural expectations of gender.

Participants recognised this as linked to broader gender norms around modesty:

“*…I think not just in Singapore, but as in just in general like men, women are put on like a different standard in terms of like modesty.*”

(Anny, 27)

Anny noted the sexual double standards that women experience are norms enforced more widely than in Singapore, highlighting the importance of such when understanding VUs. Typically, these norms result in greater restrictions on women’s sexuality and bodily autonomy (Crawford & Popp, 2003). However, others noted how gender norms and expectations may be intensified through cultural and religious norms:

“*women are more affected by men especially in today’s context where we have different racial groups… different religions… like a Muslim lady who is modest or practising their religion will feel more affected than their counterpart*”

(Jean, 32)

Jean’s recognition of the potential diversity in women’s experiences of VUs highlights the intersectional nature of victim harm, showing how gender, culture, race, and religion may collectively shape the severity and meaning of victimisation. As religious norms can further emphasise values of modesty, this can potentially intensify the sense of violation, highlighting how harm is not just psychological, but also moral and identity-based.

#### 4.2.2. Subtheme 2.2 Moralised Modesty & Responsibility

Participants often reflected on how Singaporean culture promoted conservative values of privacy and modesty, which could contribute to how VUs were perceived:

“*I think in Singapore there’s a lack of awareness on upskirting? It’s taboo to talk about it, it is as taboo as like rape or molest because anything that has to do like with sexual things…I don’t know Asians just tense up…when they hear about…any sexual things*.”

(Jean, 32)

Singapore has been described as having a “sexually repressed” culture whose conservative values can make talking about sexual issues challenging (K. P. Tan, 2003, p. 403). Jean’s reflection showed how talking about sexual offences can be uncomfortable and potentially stigmatising, as it violates cultural norms around privacy and modesty. These cultural values informed perceptions towards VUs, which could result in victim-blaming attitudes:

“*…When they’re taking escalator or stairs, ask them to be more mindful of where they stand or better still, just wear safety pants when wearing skirts. We cannot control the people who do it, even the government…because the cases are still happening, so for women to help themselves, they should do that.*”

(Mahmat, 30)

Mahmat’s perspective of VUs, embedded in gender roles and expectations, positions women as responsible for reducing their own risk of victimisation, whilst positioning men, ‘the people who do it’, as uncontrollable. Such statements reflect how self-protective behaviours are ingrained in daily routines and align with Yeoh and Yeow (1997) wherein Singaporean women reported beliefs that men have a natural inability to control their sexual urges, and so women should aid this by being modest. The conflation between modest clothing as a safety measure further evidences their construction of modest clothing as forms of safety (e.g., ‘safety pants’), which was reiterated by Sally:

“*They should consider wearing safety shorts and should be checking to make sure that no one is standing on the step right behind them*”

(Sally, 27)

Positioning women as agents who should change their behaviour to avoid victimisation can result in victim-blaming narratives, which remove the accountability of perpetrators. This supports findings by S. Tan (2023) who found that victim-blaming attitudes towards VUs in Singapore were understood through gendered expectations where women were expected to minimise their risk. Participants often reflected on the negative impact these attitudes could have on victim-survivors ability to get support or report VUs:

“*anything that’s affecting our dignity… it’s something that is embarrassing to report… Singaporeans still have that kind of mindset…that’s why…when it happens to someone, they probably try to downplay it and not… report it and make it such a big hoo ha*”.

(Haze, 33)

Haze reflected on how Singaporean culture may contribute to the silencing of VU victim-survivors. The stigmatisation associated with the sexual nature of VUs frames VUs as shameful experiences, with victim-survivors being viewed as morally compromised (lost their ‘dignity’). Their reflection that Singaporeans ‘downplay’ these incidents and should avoid making a ‘big hoo ha’ suggests that reporting VUs may be seen as an overreaction, thereby collectively discouraging reporting. Thus, suggesting culture may contribute to the tolerance and minimisation of VUs, with modesty and restraint prioritised.

The barriers cultural attitudes posed to help-seeking were recognised to be exacerbated by the lack of knowledge of legislation and support services, as well as victim-harm:

“*…Being scared not knowing what to do, not having the right resources… And not knowing if the law can even help them.*”

(Anny, 27)

Anny demonstrated how the perceived inefficacy at an institutional level (see subtheme 1.1) could result in distrust in the legal system’s capacity to deliver justice to victims of VUs, and ambiguity in navigating these processes. Such considerations reflect how IBSA is perceived more broadly (e.g., Henry et al., 2018), which, in the context of disclosure thought to elicit embarrassment and/or fear of disbelief, can create barriers to reporting and justice seeking (Lam & Lau, 2024; Ruvalcaba & Eaton, 2020) and impact true VUs statistics. This underscores the need for trauma-informed approaches to ensure VUs do not remain undetected and are addressed adequately and fairly (Fraga Dominguez et al., 2024).

#### 4.2.3. Subtheme 2.3 Harm Awareness & Minimisation

Despite believing the government did little to educate the public about VUs, most participants were aware of the harm victim-survivors could face:

“*…it’s just unfair that, you know, just one small act can cause so much mental and emotional damage…like reputationally as well if their videos go out online.*”

(Anny, 27)

By describing it as ‘unfair’ that a seemingly ‘small act’ can cause such significant harm, Anny recognises how the harm is disproportionate and likely exacerbated by the injustice victims may face. Concerns about reputational harm suggest that this may be partially caused or exacerbated by the stigma and victim-blaming responses victim-survivors may face. By commenting on the potential distributive nature of VU content, Anny recognises the potential for VUs to also result in NCSII, showing the potential for IBSA experiences to overlap. Sally similarly commented on the psychological harm victim-survivors can face:

“*I think they will feel unsafe in the streets because …if you are out and about, you might feel like, oh my gosh, like, what if this person has seen my photos before?*”

(Sally, 27)

Sally’s observation noted how victim-survivors’ sense of safety and agency may become jeopardised. She recognises the overwhelming fear victim-survivors of VUs may face when in public which could result in paranoia and hypervigilance, as reported by victim-survivors of VUs (Lam & Lau, 2024) and other forms of IBSA (Huber, 2023). This was similarly, reported by Jean:

“*If it happens to me, I will feel like my dignity will be so low. Like… I don’t have any worth in me and I wouldn’t leave the house, let alone seek a new partner…*”

(Jean, 32)

Jean’s reflection highlighted how this fear can result in victim-survivors isolating themselves and in a significant loss of trust, as reported by victim-survivors of NCSII (Huber, 2023). Their comment supports how VU victimisation might lead one to restrict their broader socialisation and undergo behavioural change; reflecting Lam and Lau’s (2024) report. Furthermore, Jean’s expression on their loss of ‘worth’ and ‘dignity’ shows how victim-survivors’ sense of self and identity may also be impacted, which could be linked to the importance of cultural norms. Although there is limited literature on the impacts of VUs specifically, these findings align with those that examined the impacts of IBSA more broadly (Bates, 2017; Hall et al., 2023); demonstrating the multifaceted consequences experienced by victim-survivors and underscoring the importance for effective recourse.

However, despite participants’ awareness of victim harm, they often reflected on how they did not believe this was recognised in broader Singaporean society:

“*They know it’s wrong and that’s it. They don’t know the consequences. They don’t know how this is going to impact the victim. They just like, OK, it’s just news… I don’t think it’s something people take seriously over here.*”

(Kelly, 32)

Kelly recognised the limited awareness of victim impact at a societal level. This disconnect between offence recognition and empathic engagement leads to VUs being understood only at surface-level; potentially leading to inadequate societal responses that further enhance the trauma and stress faced by victims (Pemberton & Mulder, 2023). To prevent minimisation and strengthen deterrence, the misalignment between acknowledging VUs as illegal acts and understanding the associated harms warrants rectification as disengagement from civic concern can result in passivity, injustice, and inequality (Anbari & Gholamian, 2016). In a Singaporean context, although visible efforts in intervening were put forth by several members of the public (Vitis, 2021b), given the upward trend and lack of local research in voyeuristic offences, these efforts are not reflective of broader public norms.

## 5. Conclusions

This study delineated the understanding and perceptions of VUs, motivations to perpetrate VUs, and the impacts thereof within a sample of young adults from Singapore. Consistent with Harper et al.’s (2021) conceptualisation of upskirting, participants attributed both sexual and non-sexual motivations to engaging in VUs, including social (e.g., technological affordance) and individual (e.g., sexual deviancy) influences. Moreover, our data—to some extent—aligned with Lister and Gannon’s (2024) Descriptive Model of Voyeuristic Behavior, in that an array of affective, behavioural, cognitive, and contextual factors were considered, by our participants, to play a key role in ones’ propensity to engage in VUs. Whilst important to note that our data are not derived from those who have committed VUs themselves, we offer unique insight to these extant models through discussion of the role of societal expectations (e.g., those pertaining to modesty and sexual taboos) and how this might facilitate harm minimisation.

VUs were broadly considered to be gendered crimes, with women the primary targets, and where blame was shifted onto the victim-survivors if they were considered to have violated societal norms of modesty and/or they had disengaged from self-protective strategies. In the context of legal reform in Singapore via the Criminal Law Reform Act 2019 (s 377BB), such efforts were perceived as insufficient and inconsistent. Socially, the majority of participants expressed how discourse pertaining to VUs and its consequences are impacted by taboos and Singapore’s conservative disposition surrounding sexual topics. With perceptions anchored in reality (Barnum et al., 2021), increased awareness, reporting, visibility, and discourse around IBSA is crucial to impact societal narratives and promote deterrence.

## 6. Limitations

Findings should be discussed in light of the study’s limitations. First, the study recruited young adult Singaporeans via social media, who were fluent in English. As such, the sample does not represent older adults, non-English-speaking Singaporeans, or those who do not use social media. Subsequently, the perspectives shared are from a narrow group of young Singaporean adults, which may not reflect the wider Singaporean culture. For example, the focus on social media discourse surrounding gender norms may be disproportionately salient due to the sample, which might have led to a more Western-aligned view, under-capturing more traditional or conservative values that may influence attitudes in older or less digitally-engaged groups. As such, future research could further explore this by accessing a more diverse sample of Singaporean nationals, or by focusing on older Singaporean adults’ perceptions, thereby enabling a more substantial understanding of victim-blaming attitudes and their interplay with societal norms.

Second, as discussed throughout, sexual topics are taboo in Singapore, meaning that we need to remain sensitive to how such views might have restricted engagement in the study as a whole, and how this might have impacted the views expressed. This might also reflect the tendency for participants to only briefly discuss mental health implications, which have been so prevalent elsewhere (Bates, 2017; Fido & Harper, 2020; Hall et al., 2023). What is also missing from this work is the voices of key stakeholders including victim-survivors (see Mclocklin et al.’s (2025) work on NCSII), perpetrators, and both law enforcement and lawyers (as in Rousaki and Fido’s (2026) work on deepfake sexual abuse). Triangulating such experiences would not only aid in understanding the contemporary positioning of VUs within a Singaporean context, but would better illuminate means of reducing perpetration and enhancing the victim-survivor experience.

Third, the interviews were conducted in English, as required by the University’s ethical procedures. While English is widely used in Singapore, conducting interviews in the participant’s non-native language may influence rapport building and the interpretative quality of the data. Marschan-Piekkari and Reis (2004) argue that cross-cultural interviews conducted in a non-native language may result in more simplified expressions or misunderstandings, even when fluent. Thus, whilst the interviews were conducted by a Singaporean national to reduce power asymmetry and foster cultural alignment, the use of English may still have influenced how participants articulated their views, which may have meant certain cultural nuances were lost. Future research may, therefore, benefit from conducting interviews in the participants’ preferred language to capture richer linguistic and cultural meanings.

## 7. Implications

By gaining insight into Singaporean norms pertaining to modesty and how this, alongside taboos around sexual topics, might influence victim-blaming tendencies, this study has several implications for addressing VUs in Singapore at different levels. For legislators, there is a clear need to improve the communication of laws and consequences pertaining to VUs, especially given the high (known) rates of perpetration in this region. For law enforcement agencies, this study has implications for how means of reporting need to be clear and easy to use, and how messaging around VUs specifically, and image-based sexual offences more broadly, should pay more attention to the victim-survivor experience. This is vital given that victim-survivors who report negative experiences disclosing sexual violence have worse psychological outcomes (Ullman, 2023), and thus, adopting a legal environment where victim-survivors feel they can trust police and be taken seriously is vital for the long-term recovery. Whilst the need for legal and police reform for VUs is not unique to Singapore, the sociocultural context in Singapore has been found to reduce the effectiveness of legal practice for other forms of violence against women and girls (Ganapathy, 2006). As such, this study also has implications for improving broader messaging surrounding VUs at a societal level to recognise the harms towards victim-survivors and to challenge victim-blaming narratives, including ones’ responsibility for preventing VUs from occurring, as touched on in this study. Subsequently, this study may also have implications for educators, who have a role in combatting the minimisation of VUs from a young age by promoting education around sexual consent, boundaries, and media literacy. Of course, there is need to work with this group specifically to best understand what is/is not currently being done to tackle this on a micro level, and the barriers educators face. Finally, given that victims of VUs can face significant harm (Lam & Lau, 2024) and the current study suggests the sociopolitical context in Singapore may restrict victims from seeking legal justice, this study also has recommendations for developing accessible victim support services, such as support groups, helplines or agencies that provide comprehensive legal resources. This comprehensive reshaping of public perceptions towards VUs is a large task, but one which can not only shift the burden of responsibility away from victim-survivors, which builds a foundation for justice and impactful rehabilitation.

## Figures and Tables

**Table 1:** Participant demographics table.

Pseudonym	Gender	Age	Race
A.H.	F	26	Malay–Indian
Kelly	F	33	Malay
Sally	F	26	Chinese
Haze	F	33	Malay
Jean	F	32	Malay
Mahmat	M	30	Malay
Naufal	M	26	Malay
Luts	M	27	Indian
Anny	F	27	Malay
Eden	M	32	Malay

## Data Availability

The data presented in this study are available upon request from the corresponding author.

## Note

1 The authors recognise the harmful discourse around the term ‘revenge porn(ography)’ to describe the non-consensual sharing of intimate images (NCSII) and its implication that said media is both generated through consensual means and shared as a direct result of the behaviour of the victim (see Fido & Harper, 2020).
